# Development and Evaluation of a Fermented Pistachio-Based Beverage Obtained by Colloidal Mill

**DOI:** 10.3390/foods13152342

**Published:** 2024-07-25

**Authors:** Anna Reale, Maria Cecilia Puppo, Floriana Boscaino, Antonela Guadalupe Garzon, Silvina Rosa Drago, Serena Marulo, Tiziana Di Renzo

**Affiliations:** 1 Institute of Food Sciences, National Research Council (ISA-CNR), Via Roma 64, 83100 Avellino, Italy; floriana.boscaino@isa.cnr.it (F.B.); serena.marulo@gmail.com (S.M.); tiziana.direnzo@isa.cnr.it (T.D.R.); 2 CIDCA-UNLP-CONICET, 47 y 116 s/n, La Plata 1900, Argentina; mcpuppo@quimica.unlp.edu.ar; 3 Instituto de Tecnología de Alimentos, CONICET, Facultad de Ingeniería Química—Universidad Nacional del Litoral, 1° de Mayo 3250, Santa Fe 3000, Argentina; agarzo@fiq.unl.edu.ar (A.G.G.); sdrago@fiq.unl.edu.ar (S.R.D.)

**Keywords:** pistachio-based beverages, lactic acid bacteria, amino acids, GABA, volatile compounds

## Abstract

The aim of the present study was to develop a fermented pistachio beverage as a plant-based alternative to milk-based drinks. For this purpose, a colloidal mill was used to finely grind and homogenize the pistachios to obtain a homogeneous consistency and prevent sedimentation. In addition, lactic acid bacteria fermentation was used to develop unique flavours and characteristics in the final product and to achieve microbiological stability for up to 30 days of storage a 4 °C. The formulated beverages were evaluated for chemical–physical characteristics (pH, organic acid production, and fructose, sucrose, and glucose content), nutritional profile (proximate composition, amino acid and GABA content), and volatile organic composition by HS-SPME-GC/MS analysis. The pistachio-based beverages were characterized by a good source of protein, fat, fiber, and minerals (mainly K and P). The colloidal mill contributed to creating a homogeneous texture and to making the nutrients readily available to the starter microorganisms, which reached concentrations above 10^8^ ufc/mL in the final products. The beverages were characterized by pronounced acidity and some by the presence of acetoin and 2,3-butanedione, volatile components associated with a yogurt- or kefir-like aroma. This innovative approach provides an alternative to traditional milk-based beverages and highlights the role of LAB in the development of nutritious and attractive plant-based beverages.

## 1. Introduction

In the last fifteen years, there was an increasing prevalence of individuals consuming a vegetarian or vegan diet in high-income countries, both in adults and young people (12–20 years) [1]. The growing popularity of this lifestyle has led to an increase in the development of many non-dairy plant-based beverages [2]. The market for these nutrient-rich beverages, high in phenols and dietary fiber [3], will reach USD 38 billion by 2024, becoming the largest plant-based food segment [4].

The best-known plant-based beverages are those made from soy, rice, almonds, and hazelnuts [5], but a wide variety of plant-based products such as nuts, coconuts, cereals, legumes, seeds, and fruits can be used in their preparation [6]. In this regard, to offer a wide range of plant-based beverages to those seeking alternatives to milk, new raw materials such as chickpea [7], amaranth [8], quinoa [9], millet [10], pine nut [11], pistachio [12], pea [13], lupine, and faba bean have recently been investigated [14]. Pistachio nuts are widely appreciated for their sensorial properties and are included as an ingredient in numerous food formulations such as ice cream, pesto, sauces, salads, and desserts or as salty snacks [15]. Bronte pistachios have a significantly higher content of phenolic compounds in the skins than in the kernel and, in addition, some compounds, such as epicatechin, quercetin, naringenin, luteolin, kaempferol, cyanidin-3-*O*-galactoside, and cyanidin-3-*O*-glucoside, are only contained in pistachio skins, giving it an excellent antioxidant activity [16]. Grace et al. (2016) [17] also suggested that the beneficial effects of pistachios could be greatly enhanced by the consumption of unpeeled kernels, as some bioactive compounds have a higher concentration in the pistachio skin than in the kernel itself.

Recent studies [3,18] have highlighted that several beverages derived from cereals and legumes present technological problems related to their processing and storage stability, such as sedimentation of larger particles, higher viscosity of the beverage, solubility, etc. Furthermore, poor sensory and flavor qualities have often been reported [18].

A common strategy used to solve many of these issues is fermentation by lactic acid bacteria and/or yeasts. This process is an ancient preservation technique that not only improves the availability of micronutrients but also has considerable advantages in improving the taste and quality of food while ensuring health properties and storage stability. Other studies have used probiotic bacteria for the development of plant-based beverages [19] showing that by-products of the cereal industry can also be successfully used for this purpose [20,21]. Several studies on plant-based fermented beverages have highlighted the ability of lactic acid bacteria to grow and improve the stability and qualitative characteristics of the final products [12,22,23,24,25]. A recent study showed that pistachios are good substrates for the development of fermented beverages due to their fermentability by lactic acid bacteria [12].

Therefore, the aim of our research was to develop an innovative pistachio-based beverage fermented with selected lactic acid bacteria belonging to the species *Leuconostoc pseudomesenteroides*, *Companilactobacillus alimentarius*, *Lactiplantibacillus paraplantarum*, *Lactiplantibacillus plantarum*, *Companilactobacillus kimchi*. A colloidal mill was considered for the preparation of the beverage as it was seen to have good application for different types of fruit, nuts, and legumes. Li et al. (2016) [26] pointed out that the colloid mill processing of mulberry generated small particle size and a disaggregated microstructure, contributing to a high content of phenolic compounds, as well as high antioxidative and α-glucosidase inhibition activities in the juice. Also, Lima et al. (2020) [27] showed that a colloidal mill allowed obtaining a cashew nut-based beverage characterized by physicochemical and microbiological stability, as well as good sensory acceptance for 60 days of refrigerated storage. Moreover, Lopes at al. (2020) [28] developed a legume-based beverage from pea, chickpea, and lupine seeds (and their blends) using several processing steps, including a colloidal mill that increased the physical stability of the beverage by reducing the size of dispersed particles. To our knowledge, a pistachio beverage made with a colloidal mill has not been investigated in depth.

For this purpose, microbiological and physicochemical analysis and volatile organic compound profiling by headspace solid-phase microextraction (HP-SPME) coupled with gas chromatography–mass spectrometry (GC-MS) were carried out to evaluate the fermentation of pistachio-based beverages obtained by colloidal milling.

## 2. Materials and Methods

### 2.1. Raw Materials

Pistachio (*Pistacia vera* L. Var. Bronte) from Bronte PDO were purchased from the Aroma Sicilia farm (Bronte, Sicily, Italy). The pistachios used in the study were harvested in 2019 and consisted of shelled, unsalted, and unroasted nuts with the following composition: 6.0% moisture, 18.1% protein, 56.1% lipids, 8.1% carbohydrates, 10.6% dietary fiber, and 1% ash. 

### 2.2. Microbial Strains

Six strains of lactic acid bacteria from the microbial culture collection of the Institute of Food Science—National Research Council (ISA-CNR, Avellino, Italy) were used in this study. The strains, previously selected for their fermentative ability, were *Leuc. pseudomesenteroides* PD4, *L. plantarum* PT1, *C. kimchi* PU2, *L. plantarum* PV2, *C. alimentarius* PG3, *L. paraplantarum* PN4 [12]. Strains were kept as frozen stocks (in 50% glycerol *v*/*v*) and routinely propagated in DeMan Rogosa and Sharpe (MRS) medium (Oxoid, Milan, Italy), pH 6.8, for 24 h at 30 °C. 

### 2.3. Pistachio Beverage Preparation

**Figure 1:**
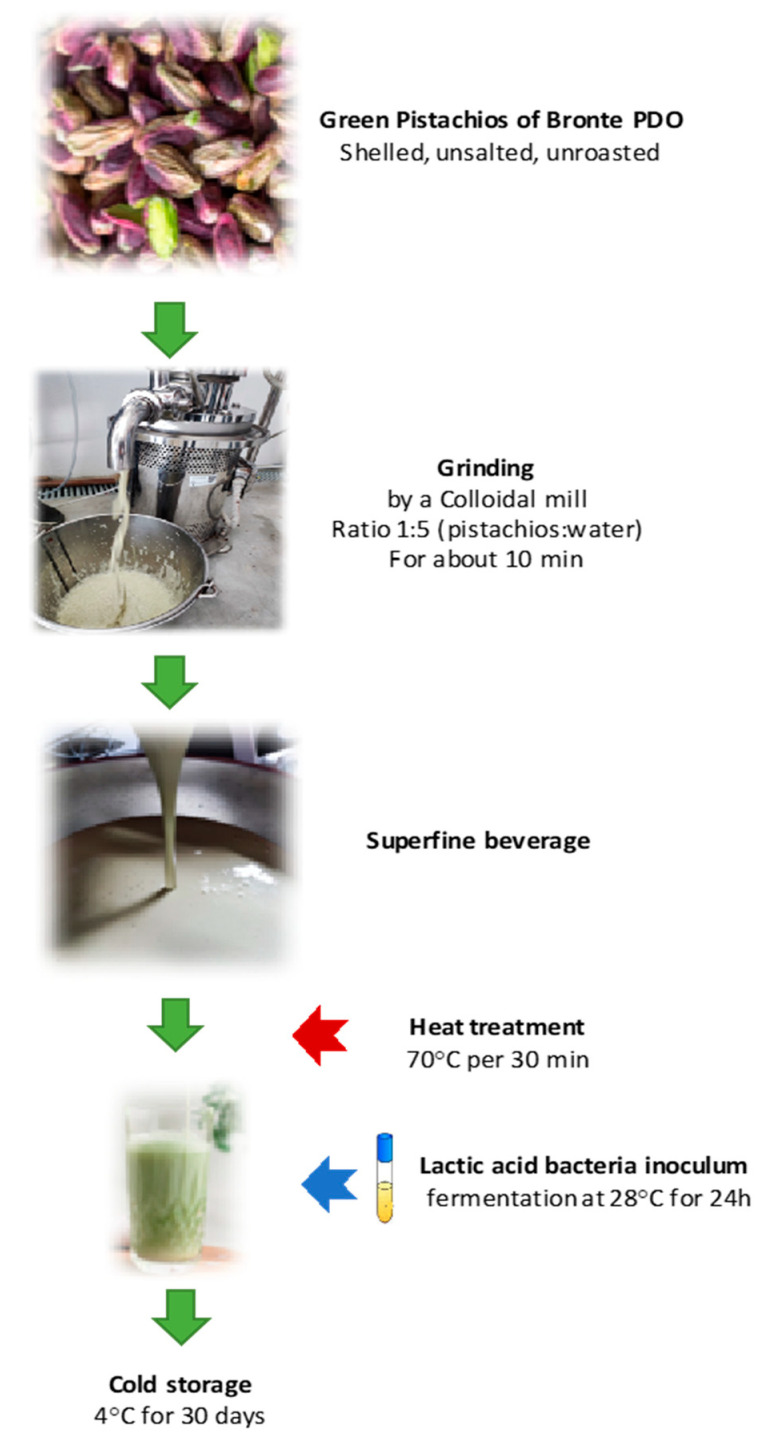
Flowchart of the preparation of the fermented pistachio-based beverage by colloidal mill.

The procedure for the preparation of the fermented pistachio-based beverage is illustrated in Figure 1. Briefly, 1 kg of shelled, unsalted, unroasted pistachios in a ratio of 1:5 (pistachios:water), was ground by a colloidal mill (Homomaster 120, S.A.R. group Galliate Lombardo, Varese, Italy) for about 10 min with recirculation at a speed of 3000 rpm. The resulting beverage was then heat-treated at 70 °C for 30 min. and cooled to 4 °C until the lactic acid bacteria were inoculated. 

### 2.4. Inoculum of Fermented Pistachio-Based Beverages

The inoculum was prepared by sub-culturing each LAB strain in MRS broth incubated at 28 °C for 16 h, followed by centrifugation at 4000 rpm for 10 min, washing of the pellet with sterile 0.9% (*w*/*v*) NaCl saline and final resuspension in the beverage at the desired concentration. Each individual strain of lactic acid bacteria was used at a final concentration of approximately 6 log cfu/mL beverage. The beverages were fermented for 24 h at 28 °C and then stored at 4 °C for 30 days. An un-inoculated beverage was used as control. Lactic acid bacteria at inoculation, after 24 h fermentation, and after 5, 12, 21, and 30 days of storage at 4 °C were verified by viable counting on MRS agar supplemented with 0.1 g/L cycloheximide (SIGMA Aldrich, Taufkirchen, Germany) at 28 °C for 72 h under anaerobic condition (Gas Pack AnaeroGen^TM^, OXOID Ltd., Basingstoke, UK).

### 2.5. pH Evaluation and Microbiological Analysis of Pistachio Beverage

Pistachio beverages were analyzed before and after heat treatment and at the end of cold storage for the presence of *Enterobacteriaceae*, fecal and total coliforms, enterococci, total mesophilic bacteria, yeasts, and molds as described by Reale et al. (2009) [29] and *Pseudomonadaceae* as reported by Belleggia et al. (2022) [30]. Furthermore, the acidifying activity of LAB cultures in the pistachio beverage was evaluated by measuring pH at time zero, after 24 h fermentation, and after 30 days of storage at 4 °C with a Medidor PH Basic 20 pHmeter (CRISON, Alella, Spain).

### 2.6. Sugar Consumption, Organic Acids, and Ethanol Production

Acetic acid, lactic acid, ethanol, sucrose, fructose, and glucose concentrations in the pistachio beverages were quantified using the RIDA^®^CUBE Assay Kits (Acetic acid RCS4226, D/L-Lactic Acid RCS4240, Ethanol RCS4340, D-Glucose RCS4140; D-Glucose/D-Fructose RCS 4160, Sucrose/D-Glucose RCS 4180, respectively) according to the manufacturer’s instructions (R-Biopharm, Darmstadt, Germany). The samples were analyzed in triplicate at time zero, after 24 h fermentation, and after 30 days of storage at 4 °C.

### 2.7. Chemical Analysis of Pistachio Beverage

Proteins were determined by measuring total nitrogen using the semi-micro Kjeldahl method (N × 6.25), and lipids (petroleum ether extract), moisture, ash, and phosphorous content were determined using AOAC (2000) [31] approved methods. Sodium and potassium were assayed by flame spectroscopy; iron, zinc, calcium, magnesium, and copper contents were measured by atomic absorption spectroscopy after dry mineralization. Before assays, the ash was removed with 10% HCl (*v*/*v*). An Analyst 300 (Perkin-Elmer Co., Norwalk, CT, USA) atomic absorption spectrophotometer was used. All determinations were performed at least in triplicate.

### 2.8. Amino Acid Profile and GABA Content of Pistachio Beverage

Amino acids were determined after derivatization with diethyl ethoxymethylenemalonate by high-performance liquid chromatography (HPLC), according to the method of Alaiz et al. (1992) [32], using D, L-α-aminobutyric acid as internal standard. The HPLC system consisted of a Perkin Elmer Series 200 pump, with a Perkin Elmer 785A UV/vis detector, equipped with a 300 × 3.9 mm i.d. reversed-phase column (Novapack C18, 4 m; Waters, Milford, MA, USA).

The content of GABA was determined according to Garzón et al. (2016) [33]. Samples (0.2 g) were extracted with trichloroacetic acid (8 g/100 mL), shaken for 60 min and centrifuged at 3000× *g* for 10 min. Supernatant (500 mL) was added to 1500 mL of borate buffer (1 mol/L, pH 9) and analyzed as explained for amino acids. 

### 2.9. Volatile Organic Compounds 

The volatile fraction of the samples was analyzed by headspace sampling using the solid-phase microextraction (SPME) technique according to Garofalo et al. (2020) [34]. In detail, for each SPME analysis, 5 g of sample was placed in a 20 mL headspace vial and 5 μL of 3-octanol (internal standard, 100 mg L^−1^ standard solution) was added. The vial was placed in a thermostatic block (40 °C) on a stirrer and the fiber was inserted and maintained in the sample headspace for 15 min. It was then removed and immediately inserted into the GC/MS injector for compound desorption. The extraction took place automatically with the multipurpose sampler of the GC/MS system. 

For the analyses, an SPME fiber coated with DVB/CAR/PDMS divinylbenzene/carboxen/polydimethylsiloxane, thickness 50/30 mm, was used (Supelco, Bellefonte, PA, USA). The SPME-GC/MS analysis was performed using an Agilent GC 7890A/MSD 5975 system with an automatic sampler Gerstel MPS2 (Agilent Technologies, Santa Clara, CA, USA). Operating conditions [25] were as follows: HP-Innowax capillary column (Agilent Technologies, 30 m × 0.25 mm ID, film thickness 0.25 μm), helium as gas carrier (flow 1.5 mL/min), and SPME injections were splitless (straight glass line, 0.75 mm I.D.) at 240 °C for 5 min, during which time thermal desorption of analytes from the fiber occurred. The oven parameters sequence was: initial temperature was 40 °C held for 1 min, followed by an increase to 100 °C at a rate of 5 °C min^−1^, and then held for 2 min, followed by an increase to 180 °C at a rate of 5 °C min^−1^, and then held for 5 min, followed by an increase to 240 °C at a rate of 6 °C min^−1^, and then held for 1 min. The injector temperature was 240 °C. The mass spectrometer operated in scan mode over a mass range from 20 to 400 amu (2 s scan-1) at an ionization potential of 70 eV. VOC identification was achieved by comparing mass spectra with the Nist library (NIST 20) and by matching the retention indices (RI) calculated according to the equation of Van Den Dool and Kratz (1963) [35] and based on a series of alkanes. Data are expressed as RAP = relative peak area (peak area of compound/peak area of internal standard) × 100 (RAP ± SD). Blank experiments were conducted in two different modalities: blank fiber and blank empty vial. The analyses were performed in duplicate.

### 2.10. Statistical Analysis

All determinations were carried out in triplicate. All results were expressed as mean ± SD (standard deviation). Mean values and standard deviations were calculated. Analysis of variance was used to determine significant differences (*p* ≤ 0.05) between means.

To determine differences between volatile compounds, one-way ANOVA and Tukey’s test were used and principal component analysis (PCA) was performed using the Tanagra 1.4 software with the dataset of VOCs.

## 3. Results and Discussion

### 3.1. Optimization of the Production of the Pistachio-Based Beverage

This study is a continuation of a previous study on the use of LAB for the production of fermented pistachio-based beverages [12]. The main differences between the two protocols used is given in Table S1.

In the previous work, a fermented pistachio-based beverage was produced using a process in which the production time of the beverage was rather long, as it was necessary to soak the pistachios for at least 5 h, grind the seeds, and filter the resulting beverage. This first protocol used resulted in the production of waste in the filtration phase of the beverage.

In the present work, it was therefore decided to use a new procedure to obtain the pistachio beverages using a colloidal mill instead of a common homogenizer, as described in Figure 1. The new production process yielded several advantages. Firstly, the use of the colloidal mill allowed for better homogenization and emulsification of the beverage, no processing waste was obtained, and the whole seed sample was incorporated into the beverage. In addition, shortened preparation time was achieved, as the previously 5 h soaking was not necessary, making the process more robust and easily scalable.

The resulting beverage was then subjected to heat treatment at 70 °C for 30 min, which was enough to remove undesirable microorganisms from the beverage [12].

Table 1 shows the microbial load values before and after the heat treatment.

The beverage was found to be contaminated with various microbial groups such as total mesophilic aerobes, *Enterobacteriaceae*, total and fecal coliforms, *Pseudomonas* spp., *Eumycetes,* and enterococci, as was also shown by Di Renzo et al. (2023) [12].

As also reported by other authors, raw pistachios are easily contaminated by molds or other pathogenic microorganisms during pre- and post-harvest practices such as harvesting, drying, hulling, and shelling [36,37]. Also, Al-Moghazy et al. (2014) [38] found high counts of aerobic mesophilic bacteria (>10^5^ cfu/g) and unsatisfactory limits of coliforms, yeasts, and molds in many pistachio nuts and pistachio-based products. In addition, Ban and Kang (2016) [39] highlighted that pathogenic microorganisms have the ability to survive for long periods in relatively dry products such as pistachios. For this reason, microorganisms were also found in the freshly produced beverage and it was necessary to heat-treat the beverage before inoculating the LAB. As shown in Table 1, the heat treatment enabled us to restore the beverage as the counts for all microbial groups were below 1 log cfu/mL, indicating the safety of the beverage prior to inoculation with the LAB.

### 3.2. Proximate Composition of the Pistachio-Based Beverage

The composition of the pistachio beverage is presented in Table 2. It resembles the composition of the nut sample used for making the drink, since the whole nut was incorporated into the beverage through colloidal milling. In fact, pistachios have a high content of lipids, proteins, and dietary fiber [40].

There are no studies in the literature on fermented pistachio beverages obtained by colloidal milling for comparison. However, Lima et al. (2020) [27] studied a cashew nut-based beverage obtained by grinding the kernel with water in a 1:10 ratio using a colloidal mill. They also added sugar at 30 g/L and treated the sample at 140 °C for four seconds. These authors reported that the colloidal grinding process and heat treatment gave stability to the beverage, preserving its physical and chemical characteristics and ensuring its microbiological quality. The beverage was characterized by lower values of protein (1.83%) and lipids (3.97%) and higher carbohydrates (5.43%) than our beverage, obviously resulting from the different raw material and dilution used in its production. As pointed out by Jeske et al. (2017) [41], who analyzed 17 different plant-based milk substitutes on the market, the nutritional and physicochemical properties are strongly dependent on the plant source, processing, and fortification.

Compared to other beverages on the market that contain <1% protein, this pistachio-based beverage can be considered a good source of protein, somewhat like milk- and soy-based beverages, which contain higher amounts of protein (around 3%) [42]. Furthermore, Hernández-Alonso et al. (2016) [43] highlighted that pistachios are richer in fiber than other nuts. In addition, this beverage was characterized by a high fat content (about 8%), but can be considered a healthy fat, as it is mainly composed of polyunsatured and monounsatured fatty acids [43].

The pistachio-based beverage was also characterized by the presence of different minerals with important nutritional and health aspects. The most abundant mineral was K, followed by P, Mg, and Ca (Table 2).

A beverage characterized by a high amount of potassium could be very attractive, especially for athletes, since potassium is an important electrolyte that regulates normal cellular function, muscle contractions, and vascular tone (blood pressure) [44]. Furthermore, the pistachio beverage also contains other important minerals such as magnesium, for which there is preliminary evidence suggesting its potential beneficial effects on reducing the risk of cardiovascular disease [45].

### 3.3. Amino Acid Profile and GABA Content of Pistachio Beverage

The amino acid profile of the fresh beverage is shown in Table 3. It was similar to that reported by Kashaninejad and Tabil (2011) [46] for the Iranian pistachio variety ‘Ohadi’.

Glu, Arg, and Ser were the main amino acids present in the beverage. Proteins in the pistachio beverage had 29.1% basic amino acids (Lys, Hys, and Arg), 28.4% acidic amino acids (Asp and Glu), 23.3% polar and hydrophilic amino acids (Thr, Cys, Tyr, Ser, and Gly), and 19.2% non-polar hydrophobic amino acids (Table 3). Pistachios also have a higher ratio of essential amino acids than most other commonly consumed nuts (almonds, walnuts, pecans, and hazelnuts), and a high proportion of branched-chain amino acids [47].

Regarding GABA, the content (9.08 mg/100 mL) of the pistachio beverage is in the range reported by Ma et al. (2022) [48] for 11 varieties of mung beans (0.77 to 16.78 mg/100 g d.b.). A balanced diet provides about 740 mg of GABA per day through the consumption of vegetables, fruits, and low-fat dairy products [49]. GABA is a non-protein amino acid with several well-known physiological functions, such as neurotransmission and the induction of hypotensive, diuretic, and tranquillizing effects [50]. Low levels of GABA have been associated with mood disorders such as anxiety, depression, insomnia, irritability, and restlessness [51]. GABA should be present in the diet, but the upper recommended intake of GABA is usually 3 g per day, with no more than 750 mg per dose.

### 3.4. Microbiological Analysis, pH, and Acid and Sugar Content of the Fermented Pistachio-Based Beverage

The fresh pistachio-based beverages were then inoculated with six strains of lactic acid bacteria (*Leuc. pseudomesenteroides* PD4, *L. plantarum* PT1, *C. kimchi* PU2, *L. plantarum* PV2, *C. alimentarius* PG3, *L. paraplantarum* PN4) previously selected according to different technological characteristics as reported by Di Renzo et al. (2023) [12].

**Figure 2:**
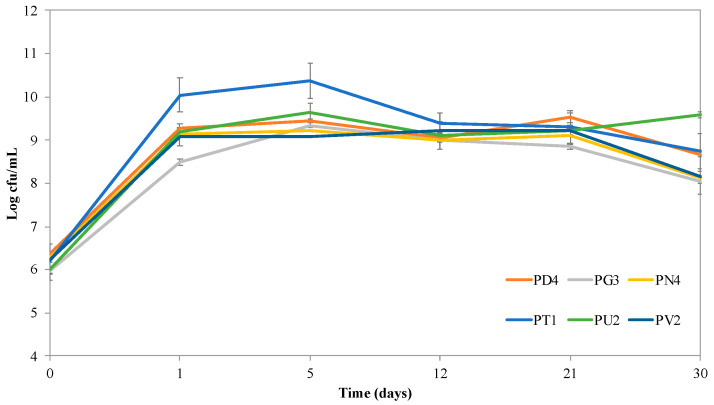
Microbial count of lactic acid bacteria in fermented pistachio-based beverages at inoculum (time zero), after 24 h fermentation (1 day), and after 5, 12, 21, and 30 days of storage at 4 °C. The labels PD4, PG3, PN4, PT1, PU2, PV2 are fermented pistachio-based beverages inoculated respectively with *Leuc. pseudomesenteroides* PD4, *C. alimentarius* PG3, *L. paraplantarum* PN4, *L. plantarum* PT1, *C. kimchi* PU2, *L. plantarum* PV2.

Figure 2 shows the microbial counts at time zero (inoculation), after 24 h fermentation at 28 °C (1 day), and during storage of the beverage at 4 °C for 30 days.

The selected LAB were inoculated at approximately 6 log cfu/mL and reached high counts after 24 h of fermentation (1 day) at 28 °C, with values ranging from 8.5 log cfu/mL (*C. alimentarius* G3) to 10 log cfu/mL (*L. plantarum* T1), demonstrating that pistachio is a good growth source for LAB.

The pistachio-based beverage at time zero had a pH value of 6.39 ± 0.09, and lactic and acetic acids and ethanol were not detected. The concentrations of sucrose, glucose, and fructose were 1.55 ± 0.08, 0.26 ± 0.02, and 0.19 ± 0.08 (g/L), respectively. The characteristics of the pistachio-based beverage after fermentation and after 30 days of storage at 4 °C are shown in Table 4.

After 24 h of fermentation, all beverages showed strong acidification, reaching pH values between 3.84 and 3.92 (PV2 and PT1, respectively). The production of lactic acid was between 7.58 g/L and 10.94 (PT1 and PN4, respectively). Acetic acid values were below 0.5 g/L in all the samples. A low amount of ethanol was found only in the sample PD4 (0.2 g/L). After 30 days of storage at 4 °C, the pH values remained more or less similar and lactic acid values remained roughly similar for PU2 and PV2 and slightly increased for PD4, PG3, PN4, and PT1. Acetic acid also increased very little in some samples after storage.

Glucose and sucrose were almost completely utilized by all the strains in the beverages after 24 h of fermentation. However, in regard to fructose, after 24 h only PG3 and PT1 strains showed a significant reduction. After 30 days of storage, all the sugars analyzed were not detected. Thus, despite cold storage, the strains continued to have fermentation activity, albeit very slowly.

### 3.5. Volatile Organic Composition of the Fermented Pistachio-Based Beverage

The fermented pistachio-based beverages were further analyzed for their volatile compound profile.

The HS-SPME-GC/MS analysis of the samples detected the presence of major and minor volatile compounds, recognized as being responsible for the aroma of these fermented beverages. A total of 23 volatile compounds were detected, belonging to aldehydes (2), ketones (4), esters and acetates (1), acids (2), alcohols (7), terpenoids (6), and benzene derivatives (1) (Table 5).

In the samples analyzed, the most detected volatile compounds belonged to ketones, terpenes, and acids, whereas small quantities of aldehydes, esters, and acetates and other substances, such as methylpyrrole, were found. Aldehydes were present in small amounts and in a few samples, the most representative being 2-methylbutanal and 3-methylbutanal.

Among ketones, 2,3-butanedione and acetoin were the most frequent in almost all the samples, with the exception of the control sample. Traces of 4-methyl-2-heptanone and gamma butyrolactone were found.

The beverages fermented with the *Leuc. pseudomesenteroides* PD4 and *C. kimchi* PU2 strains were characterized by the highest amount of ketones. In particular, the sample fermented with *Leuc. pseudomesenteroides* PD4 had the highest amount of 2,3-butanedione and acetoin, compounds that are highly valued for their pleasant buttery, fatty, and fruity notes associated with a yogurt-like aroma, kefir, or a sweet cheese flavor [34]. Samples fermented by *C. kimchi* PU2 and *L. plantarum* PV2 were also characterized by high amounts of 2,3-butanedione and acetoin. In the beverages fermented by *L. plantarum* PT1, *C*. *alimentarius* PG3, and *L. paraplantarum* PN4, acetoin was found in traces. It is known that acetoin and diacetyl are produced by LAB and contribute to butter flavor and aroma, especially in dairy products, so the acetoin production of different LAB such as *Leuconostoc* [52], *Lactoccocus lactis* [53], and *Companilactobacillus* and *Limosilactobacillus* species [54] has been investigated.

However, the production of butter aroma compounds in commercial plant-based fermented dairy alternatives (PBDA), including coconut, oat, rice, hemp, pea, hazelnut, and soy milk, was also determined by Xiao et al. (2024) [55]; these authors demonstrated significant variations in PBDA fermentation performance by using different strains. Xiao et al. (2024) [55] found different lactic acid bacteria species producing acetoin and diacetyl in different amounts, including *Leuconostoc* spp., *L*. *paraplantarum*, *L. plantarum*, as well as *E. casseliflavus*, *L. brevis*, *L. paracasei*, *L. rhamnosus*, *L. sakei,* and *P. pentosaceus*.

Furthermore, other authors [56] investigated the ability of different LAB isolates from plants to produce acetoin in the liquid fractions of brewers’ spent grain. They found that strains of *Leuconostoc pseudomesenteroides* seem to be the most suitable LAB for the production of acetoin at the highest value, with acetate as the only major by-product.

Among esters and acetates, traces of ethyl lactate were only detected in the sample fermented with the strain *L*. *paraplantarum* PN4.

Among the alcohols, ethanol, isoamylalcohol, and 1-hexanol were detected. Samples fermented with *Leuc. pseudomesenteroides* PD4 were characterized by the highest amount of alcohols, mainly ethanol. *Leuconostoc* spp., being an obligate heterolactic lactic acid fermenting bacterium, utilizes glucose as its primary source of metabolism, in addition to other sugars such as sucrose and fructose, creating ethanol, lactate, and CO_2_ as products of fermentation [57]. In addition, all the other sample beverages were characterized for the presence of alcohols such as 1-hexanol and isoamylalcohol.

Among the acids, acetic acid was the only one present in all samples and especially in the beverage fermented with *Leuc. pseudomesenteroides* PD4. Only in one sample were traces of nonanaoic acid found.

The fermented pistachio-based beverages were also characterized by the presence of high amounts of terpenoids, mailnly α-pinene, followed by δ-3-carene, limonene, β-pinene, camphene, and cymene. Among other compounds, methylpyrrole was found in all the samples.

The most representative was α-pinene, characterized by a resinous pine-like flavor, which strongly contributed to the aromatic profile of the beverages. In addition to α-pinene, discrete amounts of other terpenes, such as limonene and 3-carene, played a crucial role in defining the aroma and taste of the pistachio-based beverages. The terpenes β-pinene and cymene were found in traces.

**Figure 3:**
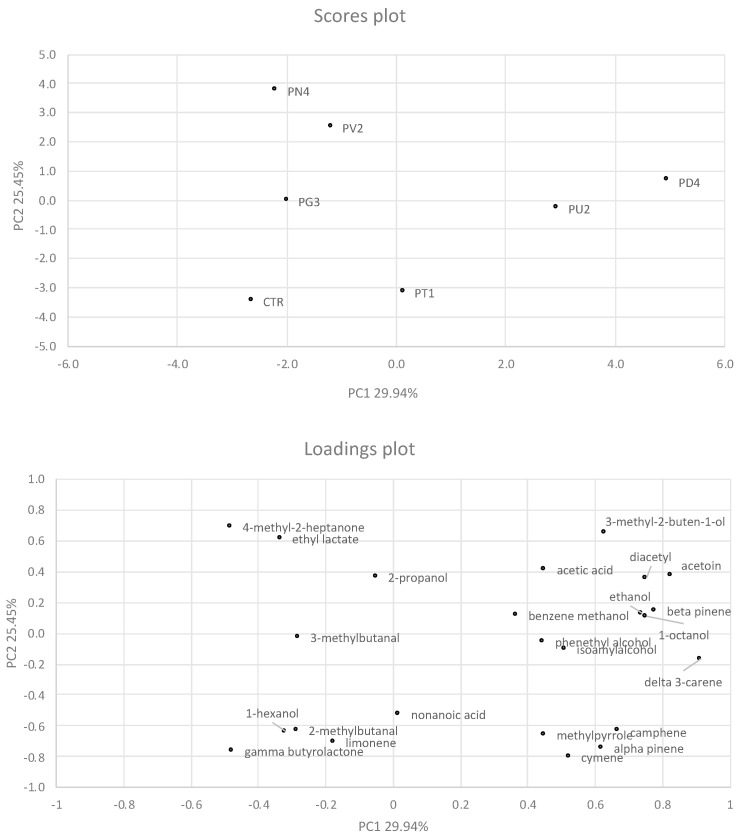
Score and loading plots of the principal component analysis (PCA) of the volatile organic compound (VOC) data set of fermented pistachio-based beverages: CTR, control; PD4, PG3, PN4, PT1, PU2, PV2 are fermented pistachio-based beverages inoculated, respectively, with *Leuc. pseudomesenteroides* PD4, *C. alimentarius* PG3, *L. paraplantarum* PN4, *L. plantarum* PT1, *C. kimchi* PU2, *L. plantarum* PV2.

The use of HS-SPME-GC/MS enabled the fermented samples to be distinguished on the basis of the aroma profile (Figure 3).

The control sample differed from all fermented beverages produced. The first two principal components (PCs) explained 55.5% of the total variance. The samples, as determined by the two PCs (factors), were located in different zones of the plane, indicating that the volatile profile of the samples was different. In particular, the sample produced with *L. pseudomesenteroides* PD4 was located in the I square of the graph, the beverages obtained with *L. paraplantarum* PN4, *L. plantarum* PV2, *C. alimentarius* PG3 in the II square, the control sample in the III square, and the samples from *L. plantarum* PT1 and *C. kimchi* PU2 in the IV square.

## 4. Conclusions

In this work, the production process of a fermented pistachio-based beverage was developed. The use of a colloidal mill resulted in a pistachio-based beverage with homogeneous textures and dispersions, and no processing waste was produced, as the whole seed was incorporated into the beverage. Moreover, shorter preparation times due to the absence of the soaking phase was achieved. The pistachio beverages proved to be excellent substrates for the growth and survival of lactic acid bacteria, which reached more than 9 log cfu/mL after 24 h of fermentation and were alive after 30 days of storage at 4 °C. Furthermore, the LAB strains used showed different performances, influencing the quality characteristics and flavor profile of the finished products. Some fermented pistachio-based beverages were characterized for the presence of acetoin and 2,3-butanedione, volatile components associated with a yogurt-like aroma, sweet cheese flavor, or kefir aroma. Furthermore, lactic acid fermentation gives the matrix the acidic sensory profile that characterizes conventional milk-based yogurts. Overall, the available evidence suggests that fermented pistachio-based beverages are a promising area of research and development in the field of plant-based foods and beverages. Further future studies will be useful to assess the sensory profile and consumer acceptability of the product.

## Figures and Tables

**Table 1:** Microbial load (log cfu/mL) of the pistachio-based beverage before and after heat treatment at 70 °C for 30 min.

	Total Mesophilic Bacteria	*Eumycetes*	*Enterobacteriaceae*	Total Coliforms	Fecal Coliforms	*Pseudomonas* spp.	Enterococci
**Fresh beverage**	5.8 ± 0.48	2.7 ± 0.05	5.5 ± 0.18	4.0 ± 0.22	3.0 ± 0.08	4.1 ± 0.32	2.7 ± 0.06
**Heat treated beverage**	<1.0	<1.0	<1.0	<1.0	<1.0	<1.0	<1.0

**Table 2:** Proximal composition of pistachio-based beverage obtained with colloidal mill.

Components	g/100 mL
Proteins (%)	4.21 ± 0.02
Lipids (%)	8.34 ± 0.29
Total dietary fiber	2.15 ± 0.20
Ash (minerals)	0.56 ± 0.02
Total carbohydrate (different from fiber)	1.93 ± 0.49
**Minerals**	**mg/L (ppm)**
Fe	7.8 ± 0.1
Zn	2.8 ± 0.0
Ca	214.6 ± 4.8
Mg	290.9 ± 7.5
Cu	1.9 ± 0.0
K	1590.5 ± 13.0
P	1265.8 ± 58.7

**Table 3:** Amino acid composition of pistachio-based beverage.

Amino acid	mg/1000 mL
Asp	35.0 ± 1.9
Glu	242.1 ± 23.5
Ser	162.6 ± 0.3
His	12.2 ± 0.4
Gly	15.1 ± 1.6
Thr	27.1 ± 2.6
Arg	248.5 ± 1.1
Ala	88.1 ± 1.9
Pro	34.6 ± 0.2
Tyr	22.6 ± 1.5
Val	17.1 ± 0.2
Met	4.8 ± 0.2
Cys	0.6 ± 0.0
Ile	11.0 ± 0.2
Leu	13.7 ± 0.2
Phe	17.1 ± 0.9
Lys	23.6 ± 2.1
GABA	90.81 ± 1.13

**Table 4:** pH, acids (g/L), and sugar content (g/L) in the pistachio-based beverages after 24 h fermentation and 30 days of storage at 4 °C.

	After 24 h of Fermentation at 28 °C						After 30 Days of Storage at 4 °C					
Strain	pH	Lactic Acid	Acetic Acid	FRU	SUC	GLU	pH	Lactic Acid	Acetic Acid	FRU	SUC	GLU
**PD4**	3.88 ± 0.08 ^a^	9.12 ± 0.81 ^b^	0.43 ± 0.03 ^a^	0.22 ± 0.02 ^a^	0.14 ± 0.06 ^bc^	0.08 ± 0.03 ^a^	3.99 ± 0.08 ^a^	10.57 ± 0.12 ^b^	0.61 ± 0.08 ^ab^	nd	nd	nd
**PG3**	3.91 ± 0.07 ^a^	7.87 ± 0.22 ^c^	0.14 ± 0.12 ^b^	0.03 ± 0.02 ^c^	0.03 ± 0.01 ^d^	0.15 ± 0.05 ^a^	3.99 ± 0.05 ^a^	9.6 ± 0.28 ^d^	0.25 ± 0.11 ^bc^	nd	nd	nd
**PN4**	3.86 ± 0.05 ^a^	10.94 ± 0.18 ^a^	0.27 ± 0.11 ^b^	0.16 ± 0.04 ^ab^	0.17 ± 0.03 ^b^	nd	3.88 ± 0.08 ^a^	11.51 ± 0.12 ^a^	0.75 ± 0.21 ^ab^	nd	nd	nd
**PT1**	3.92 ± 0.06 ^a^	7.58 ± 0.38 ^c^	0.37 ± 0.13 ^ab^	0.08 ± 0.05 ^bc^	0.09 ± 0.04 ^c^	nd	3.89 ± 0.05 ^a^	10.78 ± 0.41 ^b^	0.42 ± 0.13 ^b^	nd	nd	nd
**PU2**	3.89 ± 0.01 ^a^	9.65 ± 0.58 ^b^	0.17 ± 0.14 ^b^	0.17 ± 0.04 ^ab^	0.08 ± 0.02 ^c^	nd	3.98 ± 0.08 ^a^	9.32 ± 0.35 ^d^	0.68 ± 0.14 ^a^	nd	nd	nd
**PV2**	3.84 ± 0.08 ^a^	10.05 ± 0.18 ^b^	0.23 ± 0.05 ^b^	0.24 ± 0.06 ^a^	0.34 ± 0.01 ^a^	nd	3.99 ± 0.07 ^a^	10.15 ± 0.11 ^c^	0.75 ± 0.12 ^a^	nd	nd	nd

FRU: fructose; SUC: sucrose; GLU: glucose; nd: not detected. Different superscript letters between means within a column indicate statistically significant differences (*p* < 0.05).

**Table 5:** Volatile organic compounds (VOCs) identified in the fermented pistachio-based beverages.

RI	Compounds	Fermented Pistachio-Based Beverages							Odor *
		CTR	PT1	PD4	PU2	PV2	PG3	PN4	
	**Aldehydes**								
903	2-Methylbutanal	0.10 ± 0.00	0.13 ± 0.01	0.05 ± 0.00	Nd	Nd	0.18 ± 0.01	Nd	Fruity
910	3-Methylbutanal	0.21 ± 0.01	0.09 ± 0.00	0.25 ± 0.01	Nd	Nd	0.70 ± 0.02	0.21 ± 0.00	Almond
	**ketones**								
968	Diacetyl	Nd	3.60 ± 0.02	30.17 ± 0.31	8.58 ± 0.25	7.77 ± 0.14	11.12 ± 0.20	7.92 ± 0.02	Butter, fatty
1287	Acetoin	Nd	0.64 ± 0.01	17.19 ± 0.53	12.29 ± 0.20	11.29 ± 0.65	0.42 ± 0.03	2.38 ± 0.06	Butter, fatty
1206	4-Methyl-2-heptanone	Nd	Nd	Nd	Nd	0.16 ± 0.01	0.08 ± 0.00	0.08 ± 0.00	Fruity
1602	Gamma-butyrolactone	0.17 ± 0.01	0.12 ± 0.01	Nd	0.05 ± 0.00	0.09 ± 0.00	0.06 ± 0.00	Nd	Caramel, sweet
	**Ester and acetate**								
1316	Ethyl lactate	Nd	Nd	Nd	Nd	Nd	Nd	0.13 ± 0.01	Butter, fruity
	**Alcohols**								
934	2-Propanol	0.78 ± 0.02	0.53 ± 0.02	0.26 ± 0.01	2.31 ± 0.03	Nd	Nd	3.26 ± 0.06	Alcoholic, pungent
940	Ethanol	Nd	Nd	15.87 ± 0.61	Nd	0.60 ± 0.10	0.90 ± 0.15	Nd	Alcoholic
1237	Isoamylalcohol	0.86 ± 0.02	1.66 ± 0.01	1.35 ± 0.02	1.73 ± 0.03	1.22 ± 0.03	1.51 ± 0.04	1.15 ± 0.01	Alcoholic, fruity
1313	3-Methyl-2-buten-1-ol	Nd	Nd	0.38 ± 0.02	0.14 ± 0.01	0.19 ± 0.02	Nd	0.22 ± 0.01	malt
1320	1-Hexanol	2.76 ± 0.05	0.61 ± 0.04	0.61 ± 0.05	0.57 ± 0.03	0.40 ± 0.03	0.43 ± 0.04	0.40 ± 0.03	Sweet, green
1520	1-Octanol	Nd	Nd	0.07 ± 0.00	Nd	Nd	Nd	Nd	Green, orange
1836	Benzene methanol	Nd	Nd	Nd	0.27 ± 0.02	0.11 ± 0.00	Nd	Nd	Floral, rose
1862	Phenethyl alcohol	Nd	Nd	Nd	0.16 ± 0.00	Nd	Nd	Nd	Floral, sweet
	**Acids**								
1415	Acetic acid	0.11 ± 0.01	30.20 ± 2.37	37.77 ± 1.55	9.71 ± 0.60	33.28 ± 1.10	6.63 ± 0.5	23.33 ± 1.09	Acidic, fruity
2144	Nonanoic acid	Nd	1.17 ± 0.03	Nd	Nd	Nd	Nd	Nd	Green, fat
	**Terpene**								
1011	α-Pinene	27.65 ± 0.6	25.86 ± 0.2	29.09 ± 2.70	28.17 ± 2.21	11.04 ± 0.09	13.25 ± 0.24	7.23 ± 0.09	Fresh, pine
1046	Camphene	0.69 ± 0.04	0.70 ± 0.02	0.88 ± 0.03	0.59 ± 0.01	0.34 ± 0.04	0.28 ± 0.01	0.21 ± 0.01	Camphor
1074	β-Pinene	0.79 ± 0.01	1.10 ± 0.04	1.44 ± 0.08	2.02 ± 0.02	1.23 ± 0.07	0.97 ± 0.01	1.00 ± 0.05	Pine, resin
1108	δ-3-Carene	2.75 ± 0.35	3.16 ± 0.17	4.19 ± 0.06	3.90 ± 0.15	2.42 ± 0.06	3.12 ± 0.15	2.68 ± 0.20	Lemon, resin
1176	Limonene	3.87 ± 0.10	1.19 ± 0.01	1.38 ± 0.10	0.77 ± 0.03	0.67 ± 0.01	0.57 ± 0.03	0.35 ± 0.02	Citrus
1244	Cymene	0.55 ± 0.03	0.87 ± 0.01	0.60 ± 0.04	0.81 ± 0.07	0.14 ± 0.01	0.45 ± 0.01	Nd	Citrus
	**Others**								
1134	Methylpyrrole	3.70 ± 0.30	8.44 ± 0.18	5.06 ± 0.24	5.21 ± 0.34	2.16 ± 0.12	3.28 ± 0.37	2.68 ± 0.09	Smoky, herbal

Abbreviations: Nd = not detected; RI = retention index. Identification via comparison with RI database. Results are expressed as RAP = relative peak area (peak area of compound/peak area of internal standard) × 100 (RAP ± SD). https://webbook.nist.gov (accessed on 15 March 2024). * Based on flavornet (www.flavornet.org) and FlavorDB (http://cosylab.iiitd.edu.in/flavordb) online databases (accessed on 15 March 2024).

## Data Availability

The original contributions presented in the study are included in the article/Supplementary Material, further inquiries can be directed to the corresponding author.

## Supplementary Materials

The following supporting information can be downloaded at: https://www.mdpi.com/article/10.3390/foods13152342/s1, Table S1: Differences in the protocols used for the production of pistachio beverage in our previous (Di Renzo et al., 2023) and the current manuscript.
