# Supplementary material for: Development and Evaluation of a Fermented Pistachio-Based Beverage Obtained by Colloidal Mill
Source: Foods. 2024 Jul 25;13(15):2342. doi: 10.3390/foods13152342 (PMC11312421; doi:10.3390/foods13152342)
Supplement: Supplementary file 1 [file foods-13-02342-s001.zip › foods-3083489-supplementary.pdf]

Table S1- Differences in the protocols used for the production of pistachio beverage in our previous (Di Renzo et al. 2023) and the current manuscript.

	Protocol steps carried out in Di Renzo et al. (2023)	Protocol steps carried out in this manuscript
**Pistachios of Bronte PDO**	Shelled, unsalted, unroasted pistachios	Shelled, unsalted, unroasted pistachios
**Soaking**	Soaking in water at 25°C for 5 h	No soaking
**Grounding**	Grinding with a Thermomix for 20 min, ratio 1:5 (pistachios:hot water at 80°C)	Grinding with colloidal mill for 10 min, ratio 1:5 (pistachios:hot water at 80°C)
**Filtration**	Debris removal with double fine mesh	No filtration
**Waste**	Waste production	No waste
**Heat treatment**	70°C for 30 min	70°C for 30 min
